# Extraction of Teeth

**Published:** 1851-10

**Authors:** C. T. Cushman


### EXTRACTION OF TEETH.

### PRACTICAL THOUGHTS ON TOOTH-DRAWING;

BY C. T. CUSHMAN, D. D. S.

"It must be allowed that there is not an operation in any branch of surgery more worthy of the particular consideration of the liberal-minded and scientific surgeon than the extraction of teeth."—*Koecker.*

"The extraction of the dentes sapientiae of the under jaw is attended with more difficulty than that of any other of the teeth."—*Fox.*

"The most expert operator cannot always avoid breaking a tooth when it is brittle or hollowed out by decay, not being then able to sustain the necessary force to extract it."—*De Loude.*

I have for a long time believed that detailed reports of *diffi-*

*cult* cases of *extraction of teeth*, furnished our journals by practical dentists, would be very acceptable and *useful* to all concerned.

Reports which should particularize the age and characteristics of the patient, the situation and condition of the diseased teeth, the instruments used and methods of operating.

This operation, old as it is and more frequently required than any other in surgery, is yet often attended with unpleasant emotions both on the part of the operator and patient. Not unfrequently it is attended with mortifying *failure* and an unjust manifestation on the part of the patient or *subject*, I had better said, against the operator. This too when no imputations could properly be made against his discretion, foresight or skill.

*We* have not yet overcome the obstacles experienced in the many years of practice of Fox, Koecker, De Loude and others. The *patriarch* Fauchard's aphorism, made nearly a century and a half ago, is still in force: "It happens every day, in taking out teeth, that we meet with difficulties which cannot be foreseen."

These difficulties and failures may result from various causes, some of which I will briefly state:

1. The breaking off or crushing of the crown, owing to the destruction of its dentine by decay, or its brittleness from a loss of its vitality.

2. The fracture of a portion of the roots, owing to their tortuous shape, the density of the surrounding alveolus, or an osseous adhesion of the roots to the alveolus.

3. Inflammation and extreme irritability of the periosteum and gums.

4. Sponginess, fungous excresences, and bleeding of the surrounding gums.

5. Timidity and foolish behavior of the patient.

To illustrate the *first* and *last* named I will here relate

Case I.—A very pompous, portly, middle-aged personage, of bilio-lymphatic temperament, with short, thick, dense, yellow teeth, generally sound, applied to have a superior bicuspid extiacted. The crown was a mere shell, and much discolored, the pulp entirely dead. For *such* a tooth, and particularly *this class* of teeth in the superior jaw, I prefer a small, simple *key* with a flat, concave, moveable bolster (padded,) and hook of ample curve. (Would for such a case prefer my own so extracted.)

I accordingly prepared my key, and after suitable preparatory scarification, carefully applied it as high up as possible, the bolster being inside. A gentle turn broke it off exactly on a line with the point of application. The man jumped up in a great rage—"Sir," said he, "you've broken my tooth; I wouldn't have had it done for fifty dollars; that is no instrument to extract a tooth with; you ought not to have taken that at all"—and so utterly refused to let any further attempt, or even examination, be made! I am aware that he could find many professional endorsers for his last assertion; but, at the same time know that the result in his case, *so far*, would have been precisely the same with the forceps; and, moreover, that *after* this result the practicability of extracting his tooth was quite as favorable as when he came to me, the shell *crown* being of no avail for extracting the root.

Case II.—A servant woman applied to have a tooth extracted which had caused her immense suffering for about *six months*. Having an inherent horror of *jaw-breaking*, and an extravagant imagination that she must suffer such martyrdom in having this *particular tooth* removed—although she had had other teeth extracted without accident—she had borne with this until actually driven, in the *desperation* of her sufferings, to the only effectual means of relief.

The tooth was the inferior dens sapientia of the left side, and stood isolated; the first and second molars having been extracted a long time previously. In consequence of the loss of support which they had afforded, this tooth stood leaning very much forward and inward. It was, nevertheless, very firmly fixed in the jaw (as this class of teeth are often found to be, to a wonderful degree) as were likewise the remainder of her teeth, she being a robust woman. The inner side of the tooth was decayed below the edge of the alveolus; the outer side was remarkably *rounded* and irregular.

Wishing and rather prefering to use the forceps for the extraction of teeth in nearly all practicable cases, I first applied the Harris' Molar Forceps—an excellent instrument for its purpose—in the hope of forcing it down on the inside, low enough at least to make a *fulcrum* of that beak, which would enable me to raise and turn the tooth in that direction with the other. But a painful trial, and a *crushing* of the inner side, only confirmed my fears and convinced me of the *impossibility* of succeeding with this instrument.

I next had recourse to the *key*, which seemed likely to be the most efficient means. The bolster was wrapped, a suitable hook selected and carefully applied to the *outer* side of the tooth, which offered ample strength for the purpose; but I could not possibly make it retain a hold on that peculiarly rounded surface, although I was aided by an assistant, so that I had both hands free to make the application. I will here observe that the tooth had not yet attained its full altitude through the gums. Here were two trials made which really effected nothing, more than augmenting the patient's pain and fears. I merely tried to see if the hook could be applied to the *inner* side with any hope of success; the posterior part of the crown there presenting a small portion that was comparatively sound. But the well-known difficulties of applying the key so far back in the mouth soon gave me to see that this design was hopeless.

I next tried the ordinary *fang forceps*, whose beaks are slim and have a simple curve, in the hope of applying them *obliquely*, one beak resting upon the hold I last had in contemplation, the other in a line opposite on the outer side. Another crushing of the softened lingual side, which produced additional pain without effecting *anything* toward its removal, told the failure of *this* scheme.

I next directed my assistant, standing on her left side, to endeavor to apply the same instrument from his position, to draw back the angle of the mouth, and apply one beak nearly

*behind* the tooth, the other in a line opposite. I have sometimes extracted difficult teeth by this kind of *cross application*, but nothing could be effected by this trial from the impossibility of forcing down the beak in front sufficient to obtain a hold.

The tooth stood, as I before stated, in a position leaning much forward, and was not fully developed in its growth; indeed I have never seen another such case.

I then took my "*langue de carpe*," or elevator, and my position on the *left* side of the patient. Resting the rounded surface of the instrument upon the anterior edge of the *alveolus*, I thrust the point obliquely downwards into it, and, with a vigorous turn of the hand, I raised the tooth almost entirely out by a single movement! so far that it was easily picked out by the forceps. The whole operation, by her own admission, caused not so much pain to the patient, as had been previously given by the repeated trials, which only resulted in failure. It was not attended with any fracture of the alveolus or unusual laceration of the gum that I could discover.

Here, at last, was the *only* way, I am satisfied, in which this tooth could have been extracted under the circumstances.

I have the handle of my elevator pierced crosswise, with two holes 5-16 of an inch in diameter, bored *obliquely*, to admit a cross-piece about three inches long. These holes intersect each other, so that the cross-piece when inserted either way stands at an angle of about sixty degrees with the shaft. This piece is to prevent the handle from slipping in the hand; and, of course, gives much greater power, a power that is frequently absolutely necessary to a prompt and effectual execution. It is made to fit the hole closely and slide out easily. I fix it in according to the direction in which I wish to turn the instrument, having the end which rests upon the joint of the index finger always nearest to the point of the instrument. But for this addition I should not have been able, in the foregoing case, to overcome the resistance of the tooth.

When extracted the roots were curved backward, and presented the most extraordinary case of *exostosis* I ever saw; they showed ample cause for the patient's intense suffering and for the difficulty of extracting the tooth.

I am well aware that there are writers who assert confidently and without exceptions, that the *forceps* are the *only* instruments proper and efficient for extracting *all* teeth and roots, and that with these in the hands of a *competent* operator success is "*always*" certain.

If this proposition is incontrovertible and *infallible*, then there is certainly a lamentable want of competency, not only among the dental profession generally, but including the medical as well as all *amateurs* who essay in the operation in question.

As a recent author very justly observes:* "Surely either extraction must be performed when the tooth might be saved by stopping, or the operators must be very fortunate in their patients, or else they must each have large cabinets of broken teeth."

And if these writers are able to *demonstrate*, practically, the correctness of their assertions I can truly say I would like to *see them do it.* I would be highly gratified as well as relieved to have them assume entirely that portion of my own office practice which is furnished by our *negro population*—a race whose violations of hygienic laws are lamentably exhibited in the soft structure and dilapidated condition of their teeth—who will seldom consent to the extraction of a tooth until its crown is mostly destroyed, its roots softened, and its pulp and membranes have repeatedly passed through the successive stages of inflammation, resolution and suppuration, and have got to that condition in which they are no longer tolerable to the *touch*, say nothing of *feeling*. Until I can witness such demonstrations on such subjects, I must be permitted to doubt. Until I shall see one promptly remove firmly articulated fangs, which are only surmounted by the softened shell of a crown reduced to a mere *rim*, and whose membranes are inflamed to that degree that the contact of an instrument is like striking a dagger

*Surg. Mech. and Med. Treat. of the Teeth, page 139.

to the heart, I must doubt. Until I see them extract such teeth, when the surrounding gums are turgid and fungous with inflammation and pour a deluge of blood upon the field of attack, and the patient objects to more than *three* trials, I must doubt. Until such challengers shall exhibit to me their *infallible* triumphs in these cases, I shall not hesitate to say that in many such which I have seen, to extract a tooth with *any* instrument, is not only a practical but a physical *impossibility;* I mean of course the patient to be regarded as a *living*, organized being. If he were simply a wooden statute I doubt not but success would crown every individual effort. Under what other metamorphose could we think of adopting the method proposed, to make a "crucial incision" in the gum as nearly as possible to the apex of the root, dissect the gum from the bone, which cut away with the point of a "strong knife" till an opening be made into the alveolar cavity and the apex is exposed, then introduce an elevator and force it out.

Although this is recommended in some such cases as I have instanced, by that high authority, Mr. Bell, who can contemplate it with feelings of toleration? Who is willing to adopt, or to *have* adopted, the plan of M. Begin, to apply a sharp-pointed hook, attached to a turn-key, directly *on the gum*, high upon the root and opposite its center.*

Talk not of "improved instruments" effecting easy triumphs in *every* case. The inventor of a *double claw* for the turnkey, regarded and claimed consideration for it as a great improvement;† and the various "attachments" and modifications of the primitive instruments for this operation alone would and *do* form a perfect *museum* in almost every cutler's shop.

But practical experience shows that the more *simple* the instrument the better it is for this purpose. My own *boutique* is somewhat extensive and embraces the approved instruments for the operation under consideration. And yet there are not a few aching teeth and "*snags*" which come to me in the course of

*Vide Desirabode's "Complete Elements," p, 304.

†"Dental Practice," p. 71.

a year that I cannot remove wholly, with all *reasonable* endeavors on my part and an equal amount of fortitude and patience on that of their possessors.

Some four years ago a young man came to me, at midnight, in great agony, caused by the first inferior molar on the right side. *Several times* did I exert as much of my bodily strength upon that tooth, with the *forceps*, as I dared to do; and without *loosening* it one atom as I could perceive. I then quit.* The tooth gradually got easy, though its nervous connection could not have been broken and I believe it is yet standing where it *grew*. This was an uncommon case but not my only one of the kind.

Many such teeth as have decayed away or been broken off I see again six months or a year afterward; when, from the more favorable circumstances, viz., freedom from *inflammation*, the shrinking of the gums, which leaves the roots more exposed to view, and from their having become somewhat detached and elevated by the natural process, I am enabled to remove them with but little trouble or pain. What is the sense, for instance, of proposing the *screw* or any modification of it for a root whose periosteum is in that state of irritability that the slightest pressure on it causes *excruciating* pain? Is there a subject who could *live* under the operation of drilling out such a fang and the application of the instrument? If so they would undergo one of the longest and most painful operations in all surgery.

I hope it will not be forgotten that I have been considering *extraordinary* cases of extraction, such as I desire to see practically treated of by writers more in detail. The operation with me *generally*, I have reason to believe, is as expeditious and *easy* with myself and patient as in the practice of others. In my confession of fallibility I must include many operators whose practice and admissions are known to me. But if I have

*I have an anomalous, pathological (presented) specimen of an *extracted* tooth, which serves me as a perpetual monitor against the exercise of *unlimited* physical force in this operation.

confessed *too much* when I say for myself and profession that we do not, *cannot* at one visit get out entire *all* aching and diseased teeth which come to us for extraction, I trust its no libel in any other sense than the *legal* one, "the greater the truth," &c. And if I am altogether "behind the age" in this particular "line," and you can "show me the man" that *never* fails, in *any case*, with the *forceps*, I would be glad to sit at his feet, as did Paul at Gamaliel's, and learn.

*October 1st*, 1851.

